# Room scatter effects in Total Skin Electron Irradiation: Monte Carlo simulation study

**DOI:** 10.1002/acm2.12039

**Published:** 2017-01-19

**Authors:** Alexander Nevelsky, Egor Borzov, Shahar Daniel, Raquel Bar‐Deroma

**Affiliations:** ^1^ Division of Oncology Rambam Health Care Campus Haifa Israel

**Keywords:** dosimetry, Monte Carlo simulation, Total Skin Electron Irradiation

## Abstract

**Purpose:**

Total Skin Electron Irradiation (TSEI) is a complex technique which usually involves the use of large electron fields and the dual‐field approach. In this situation, many electrons scattered from the treatment room floor are produced. However, no investigations of the effect of scattered electrons in TSEI treatments have been reported. The purpose of this work was to study the contribution of floor scattered electrons to skin dose during TSEI treatment using Monte Carlo (MC) simulations.

**Methods:**

All MC simulations were performed with the EGSnrc code. Influence of beam energy, dual‐field angle, and floor material on the contribution of floor scatter was investigated. Spectrum of the scattered electrons was calculated. Measurements of dose profile were performed in order to verify MC calculations.

**Results:**

Floor scatter dependency on the floor material was observed (at 20 cm from the floor, scatter contribution was about 21%, 18%, 15%, and 12% for iron, concrete, PVC, and water, respectively). Although total dose profiles exhibited slight variation as functions of beam energy and dual‐field angle, no dependence of the floor scatter contribution on the beam energy or dual‐field angle was found. The spectrum of the scattered electrons was almost uniform between a few hundred KeV to 4 MeV, and then decreased linearly to 6 MeV.

**Conclusions:**

For the TSEI technique, dose contribution due to the electrons scattered from the room floor may be clinically significant and should be taken into account during design and commissioning phases. MC calculations can be used for this task.

## 1 Introduction

Total Skin Electron Irradiation (TSEI) is one of the best treatments of malignant skin diseases, such as Mycosis Fungoides (MF) and Cutaneous Lymphomas.^1^, ^2^, ^3^, ^4^ The goal is to treat the entire surface of the skin with a relatively uniform dose, e.g., ± 10%.^5^ Since treatment dose should be restricted to a shallow depth, low energy electrons are commonly used for TSEI. Over the years, different techniques have been developed for TSEI; they are described in the AAPM Report N.23 “Total Skin Electron Therapy: Technique and Dosimetry”.^6^ However, a majority of institutions employ the Stanford technique, developed by Karzmark and colleagues,^7^ or its modifications. This technique is characterized by the use of an extended SSD and dual‐field approach in which the large electron fields are angled by approximately ± 20 degrees from the horizontal axis. In order to achieve uniform dose coverage of the whole skin surface, a patient stands in six different positions relative to each of the two large angled TSEI beams (anterior, posterior, and four lateral obliques).

The use of large electron fields coupled with the dual‐field approach produces many electrons scattered from the treatment room floor and ceiling, which might contribute to skin dose and distort dose distribution. However, no investigations of the effect of scattered electrons on skin dose in TSEI treatments have been reported. For such an investigation, Monte Carlo (MC) simulations could be used. We recently reported our experience in MC modeling of the TSEI treatment (8) and showed that MC calculations could be a promising tool for further studies of dose distribution calculations in TSEI.

The purpose of this work was to study the contribution of room scattered electrons to skin dose during TSEI treatment, using MC simulations with a validated model from our previous research.

## 2 Methods

6 MeV and 8 MeV beams from the Elekta Precise linac operated in High‐Dose‐Rate‐Electron (HDRE) mode (dose rate about 30 Gy/min at the isocenter) were modeled for the use in MC simulations.

The skin dose from one field during TSEI treatment (either horizontal or rotated by dual‐field angle) was assessed by calculating the dose at 1 mm depth at a treatment distance of 400 cm.

All MC simulations in this study were performed with the EGSnrc code^8^ for coupled electron and photon transport. BEAMnrc^9^ is an EGSnrc‐based package that allows for the simulation of radiotherapy treatment units using predefined component modules (CM). The BEAMnrc code produces phase‐space files containing all the necessary information characterizing every particle at a specified scoring plane. The phase‐space data can then be used as an input to calculate dose distributions in water phantoms or CT‐based phantoms using the DOSXYZnrc^10^ code.

Details of our linac simulation have been described preciously.^11^ Briefly, the incident electron beam parameters (energy spectrum, FWHM, mean angular spread) were adjusted first to match the measured data (PDD and profile) at SSD = 100 cm for an open field. These parameters were then used to calculate dose distributions at the treatment distance of 400 cm.

**Figure 1:**
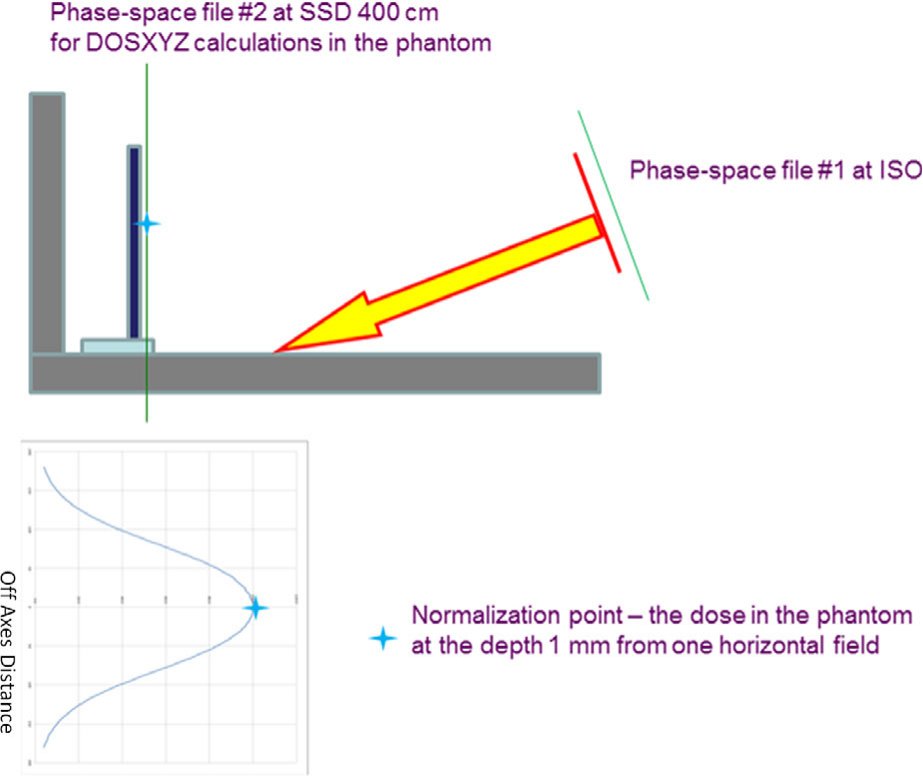
Schematic representation of the simulation geometry and dose normalization point. The star symbol (normalization point) represents the same point in both pictures.

Two phase‐space files were used in MC simulations. The first phase‐space file was generated using BEAMnrc code in a plane at SSD = 100 cm. For calculations at the treatment distance, the phase‐space file at SSD = 100 cm was used as a source within the BEAMnrc code, and a second phase‐space file was created at SSD = 400 cm. For the dual‐field, the phase‐space file at SSD = 100 cm was first rotated by the dual‐field angle at the isocenter plane and used as a source for the creation of the second phase‐space file at SSD = 400 cm (Fig. 1).

The second phase‐space file was then used in the DOSXYZnrc code for dose calculations at SSD = 400 cm in a water phantom. In large geometry conditions such as those encountered in TSEI treatment, the particle density in the second phase‐space file is much lower than in the phase‐space file at the isocenter. In order to achieve acceptable level of statistical uncertainties (3% in region of interest near the floor), large voxel size of 5 × 3 × 0.2 cm^3^ was selected (5 cm horizontally and 3 cm vertically). The splitting variance reduction technique was applied to improve dose calculation efficiency with the splitting number of 128 for photons and electrons. Following several published studies,^12^, ^13^, ^14^ the cutoff energies P_cut_ and E_cut_ were set to 0.01 MeV and 0.7 MeV, respectively. For all simulations, the boundary crossing algorithm was PRESTA‐I and the electron step algorithm was PRESTA‐II. The user adjustable values for other parameters were set at their default values.

The floor was modeled within BEAMnrc using the JAWS module. LATCH variable was used to track electrons history and calculate dose profile with and without electrons scattered from the floor.

In our room geometry, the ceiling is situated much farther from the patient than the floor, (the ceiling is about 1 m from a patient head while the floor is about 30 cm from a patient feet), therefore, only floor scattering was considered in this work.

No degrader or base system on which a patient stands on was included in the simulations.

All MC calculated vertical dose profiles were normalized to the maximum dose from one horizontal field (gantry angle 90°) at 1 mm depth (Fig. 1). The influence of floor material on the contribution of floor scatter was investigated by dose profile calculation for several materials: water, concrete, PVC, and iron (Fe). These dose profiles were calculated with the angle 17°, as it was found to be the optimal dual‐angle in our center. In order to study the influence of dual‐angle on the contribution of floor scatter, calculations for two additional angles (16° and 18°) were performed. In addition, the spectrum of the scattered electrons was calculated to assess their penetration ability.

Measurements of dose profile were performed in order to verify MC calculations, with the dual‐angle 17° and concrete as a floor material.

Measurements for beam characterization were performed at 100 cm SSD in the HDRE mode. The depth dose along the central axis and lateral profiles were measured in a water phantom (MP3, PTW, Freiburg, Germany) using the EFD electron diode (Scanditronix, Uppsala, Sweden). Output in terms of Gy per MU was measured in the same water phantom using a plane‐parallel Roos PTW ion chamber (PTW, Freiburg, Germany).

At the treatment distance, the measurements were performed in a RMI‐457 solid water phantom (GAMMEX RMI, Middleton, WI, USA) using the Roos chamber. The vertical profile was measured at 1 mm depth by shifting the solid water phantom housing vertically.

## 3 Results

**Figure 2:**
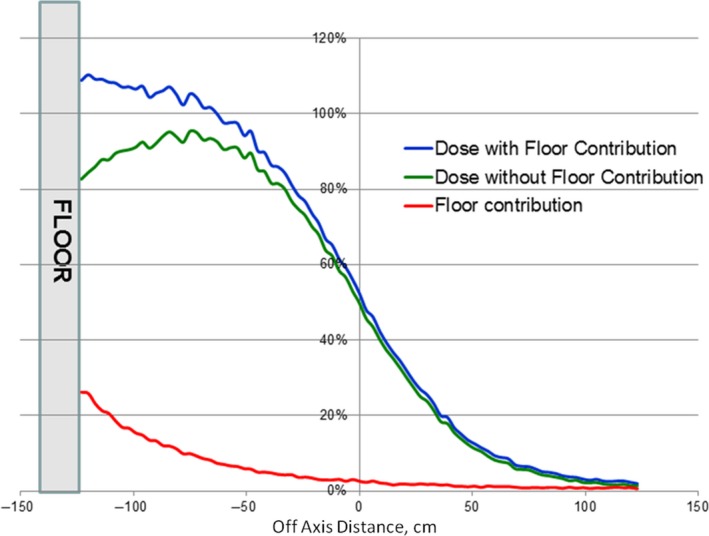
Calculated total dose, direct dose and floor contribution profiles (energy 6 MeV, gantry angle 17°, concrete as a floor material).

Figure 2 presents the vertical profile for the total dose, dose without floor scatter (particles scattered from the floor are not accounted for), and the floor scatter contribution calculated for 6 MeV beam, gantry angle of 17°, and concrete as the floor material.

**Figure 3:**
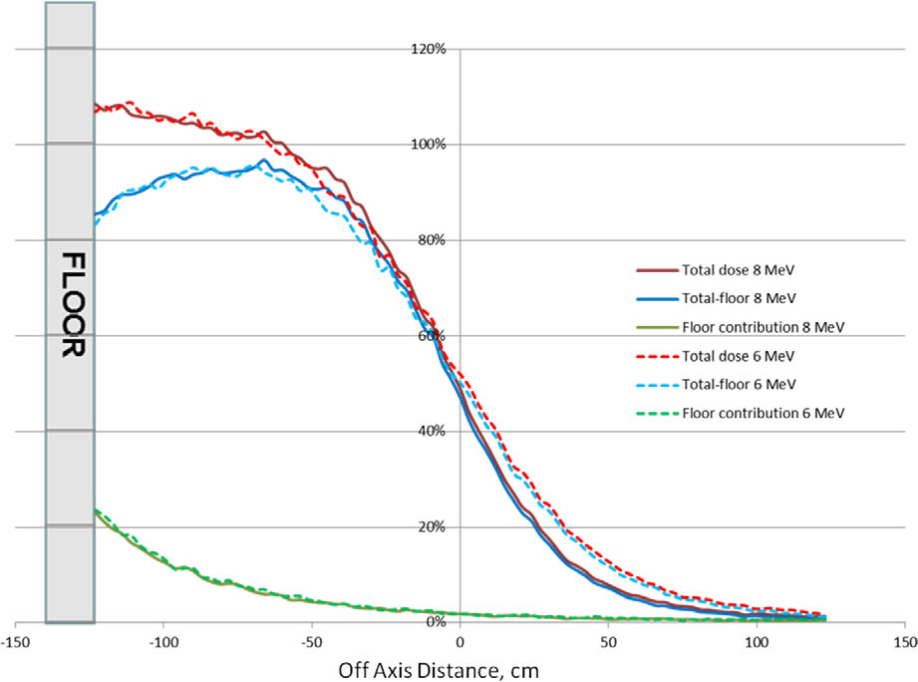
Beam energy dependence. Floor scatter calculated for 6 MeV and 8 MeV beams is shown.

Floor scatter dependence on the beam energy is shown in Fig. 3, where vertical dose profiles for 6 MeV and 8 MeV beams are presented.

**Figure 4:**
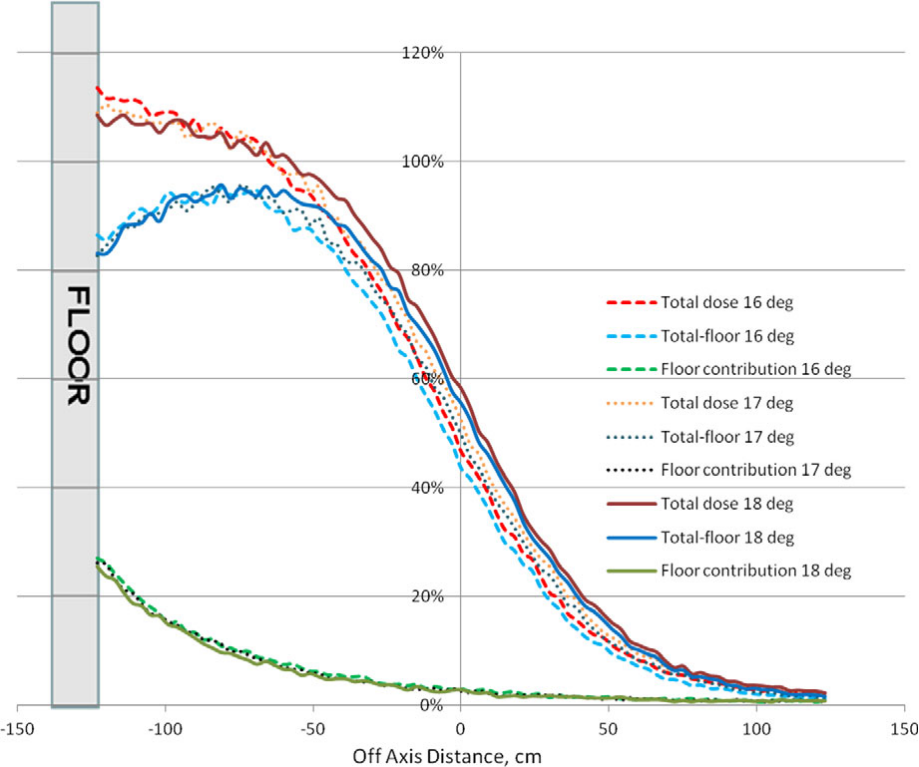
Gantry dual‐angle dependence. Floor scatter calculated for gantry angles of 16°, 17°, and 18° is shown.

Figure 4 presents dose profiles for gantry angles of 16°, 17°, and 18°, illustrating floor scatter dependence on the dual‐field angle.

**Figure 5:**
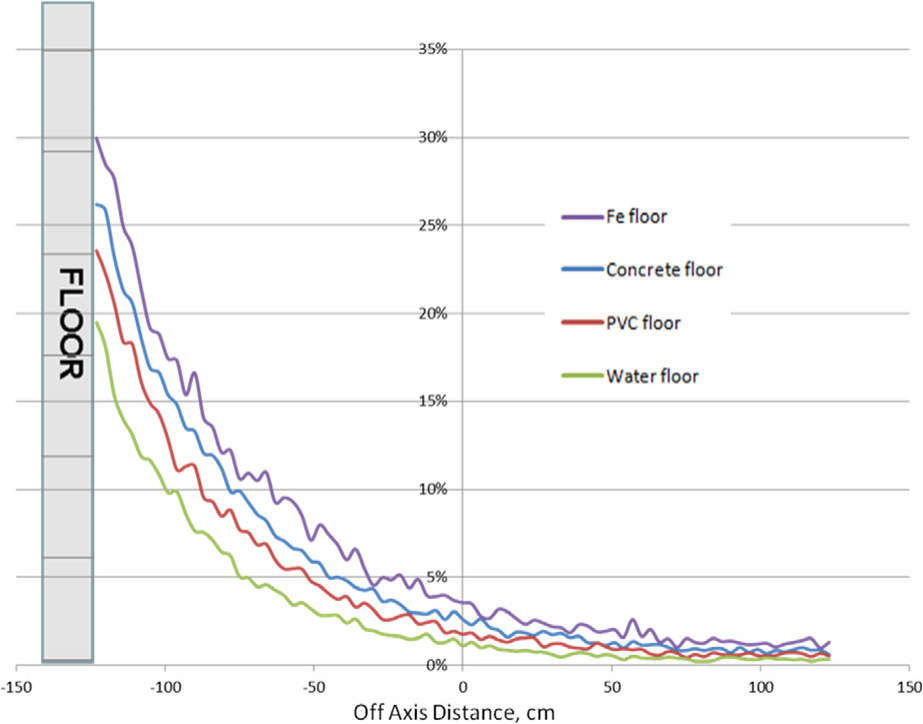
Floor material dependence. Floor scatter calculated for four different floor materials (iron, concrete, PVC, water) is shown.

Floor scatter contribution calculated for 6 MeV beam, gantry angle 17°, and four different floor materials is shown in Fig. 5.

**Figure 6:**
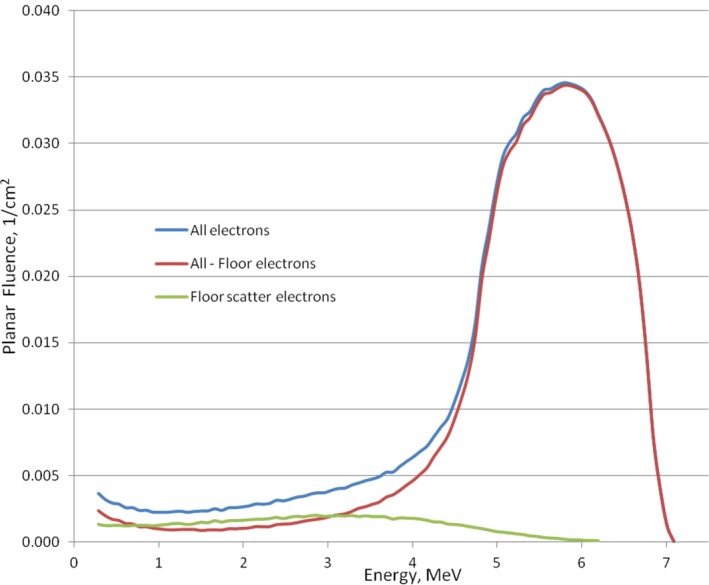
Spectrum of all electrons in the phantom, direct electrons, and electrons scattered from the floor.

Figure 6 presents a spectrum of the scattered electrons at SSD = 400 cm depth calculated for 6 MeV beam, concrete floor, and gantry angle 17°.

**Figure 7:**
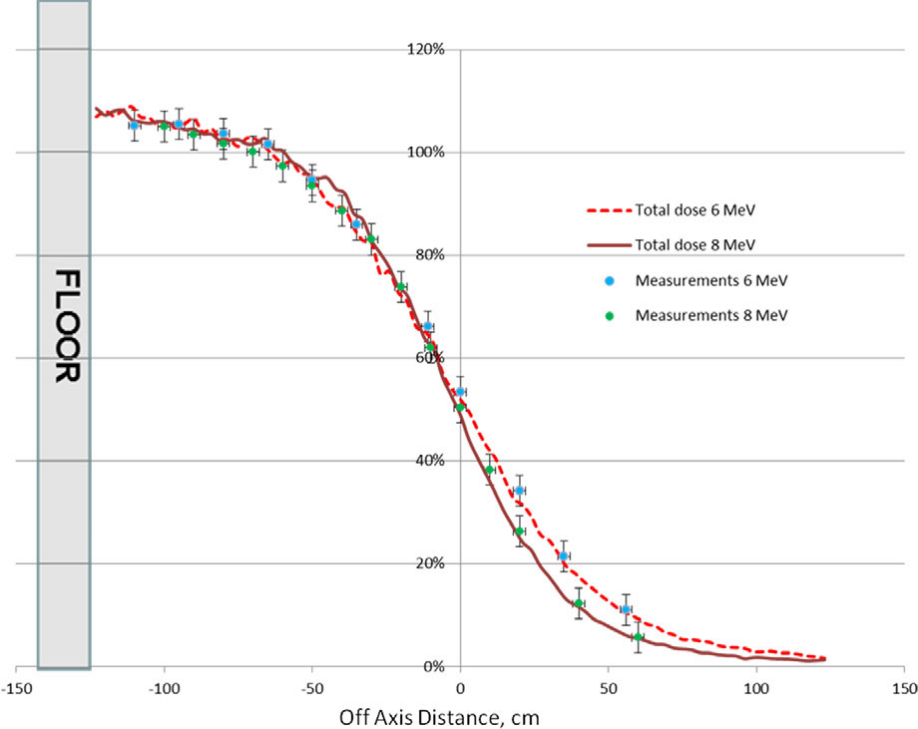
The results of dose verification measurements for the total dose performed for the 6 MeV and 8 MeV beams.

The results of dose verification measurements for the total dose performed for the 6 MeV and 8 MeV beams and gantry angle 17° are shown in Fig. 7.

## 4 Discussion

Floor scatter contribution was found to be more than 20% near the floor, decreased to about 10% and 5% at the distance of 50 cm and 100 cm from the floor, respectively.

Although total dose profiles exhibited slight variation as functions of beam energy and dual‐field angle, no dependence of the floor scatter contribution on the beam energy or dual‐field angle was found.

Floor scatter dependency on the floor material was observed (at 20 cm from the floor, scatter contribution was about 21%, 18%, 15%, and 12% for iron, concrete, PVC and water, respectively).

The spectrum of the scattered electrons had a distribution which was almost uniform between a few hundred KeV to 4 MeV, then decreased linearly to 6 MeV. One can conclude from this spectrum that the scattered electrons are much less penetrative and their dose contribution will be mostly at the phantom surface or patient's skin.

Dose verification measurements for the total dose were in good agreement (less than 3%) with the MC calculations for both energies.

Only floor scattering was considered in this work as, in our room geometry, the ceiling was situated much farther from the patient than the floor. However, the same approach may be realized for modeling of ceiling scatter, and the conclusions drawn for the floor scatter are relevant for the ceiling scatter as well.

The presence of a patient base and of a degrader which are normally used in TSEI treatments are expected to influence the scattered dose. However, these components were not included in simulations at this stage of the project and this investigation will be part of the further work.

## 5 Conclusions

For the TSEI technique, dose contribution due to the electrons scattered from the treatment room floor and ceiling may be clinically significant and should be taken into account during design and commissioning phases. MC calculations can be used for this task.

## Conflict of Interest

The authors have no conflict of interest.
